# Portal vein thrombosis and food protein-induced allergic proctocolitis in a premature newborn with hypereosinophilia: a case report

**DOI:** 10.1186/s12887-021-02510-9

**Published:** 2021-01-23

**Authors:** Cheong-Jun Moon, Tae Hee Kwon, Hyun-Seung Lee

**Affiliations:** 1 Department of Pediatrics, St. Vincent’s Hospital, College of Medicine, The Catholic University of Korea, Seoul, South Korea; 2 Department of Radiology, CHA Gangnam Medical Center, CHA University School of Medicine, Seoul, South Korea; 3 Department of Pediatrics, CHA Gangnam Medical Center, CHA University School of Medicine, 569 Nonhyon-ro, Gangnam-gu, Seoul, 06125 South Korea

**Keywords:** Eosinophilia, Food protein-induced allergic proctocolitis, Portal vein thrombosis, Premature newborn, Case report

## Abstract

**Background:**

Peripheral blood eosinophilia is identified in numerous medical conditions associated with allergic, infectious, and inflammatory processes mostly as reactive eosinophilia with or without tissue eosinophilia. In hospitalized neonates, eosinophilia is common with an inverse relationship with gestational age and occurs solely as mild eosinophilia in the majority of cases. In the literature, eosinophilia has been proposed as a possible risk factor for venous thromboembolism. However, few reports are found on thromboembolic events including portal vein thrombosis (PVT) associated with eosinophilia in the newborn period.

Neonates, particularly preterm infants, are vulnerable to thrombosis due to the immature and developing hemostatic system with little reserve capacity, which occurs as catheter-related thrombosis in most cases.

**Case presentation:**

A male newborn at 34^+ 5^ weeks’ gestation presented with a left portal venous thrombus and hematochezia after initial cow’s milk feeding in the setting of blood hypereosinophilia for a prolonged period of time without central venous catheterization. The infant was diagnosed with PVT and food protein-induced allergic proctocolitis (FPIAP) and showed complete resolution of the conditions with expectant management with food avoidance, including the normalized eosinophil count.

**Conclusions:**

Our experience suggests that in the setting of hypereosinophilia with a prolonged duration in premature neonates, FPIAP should be suspected in case of hematochezia in otherwise healthy infants, and considering the increased thrombotic risk by the hypereosinophilia and premature newborn status, evaluation for neonatal thrombosis may be needed, including PVT with the potential risk for the more serious, but uncommon, late complications encompassing portal hypertension.

## Background

Peripheral blood eosinophilia is common in hospitalized neonates as evidenced by the inverse relationship between the occurrence and severity of eosinophilia and gestational age and the reported incidence of 75% in premature infants less than 27 weeks’ gestation [1–3]. Eosinophilia in neonates has been defined as an absolute eosinophil count ≥700 cells/mm^3^, and subclassified as mild (700–999 cells/mm^3^), moderate (1000–2999 cells/mm^3^), or severe (≥3000 cells/mm^3^) [1]. Eosinophils, as myelopoietic effector cells residing in various tissues including the airways, skin and gastrointestinal mucosa, have functions such as host defense and immune modulation via secretion of intracytoplasmic granules containing cytotoxic molecules, cytokines and chemokines, which can result in end-organ damage [4–7]. In neonates, although most of neonates with eosinophilia present solely with mild eosinophilia [2], previous reports have shown various associated conditions with eosinophilia including food hypersensitivity encompassing cow’s milk allergy (CMA) and food protein-induced allergic proctocolitis (FPIAP), drug reactions, infection/sepsis, necrotizing enterocolitis, bronchopulmonary dysplasia, hypereosinophilic syndrome, and eosinophilic leukemia [8, 1–3], with some evidence of elevated eosinophil counts as a predictive marker of atopic disease development [9]. Furthermore, blood eosinophilia has been proposed as a possible risk factor for venous thromboembolism in prior reports showing possible links between eosinophilia and a prothrombotic state or thrombosis [10–14]. However, few reports are found on thromboembolic events including portal vein thrombosis (PVT) associated with eosinophilia in the newborn period.

Neonates, particularly preterm infants, have factors elevating thrombotic risk including the immature and developing coagulation and fibrinolytic systems with little reserve capacity, small vessel diameters, and numerous affected disorders that can disrupt hemostatic balance [15, 16]. Most (up to 94%) of neonatal thrombosis cases have been reported to be associated with indwelling central venous catheters, including umbilical catheters [16].

Herein, we report a rare case of PVT and FPIAP in the setting of blood eosinophilia for a prolonged period of time in a premature newborn with no history of umbilical venous catheterization (UVC).

## Case presentation

**Fig. 1:**
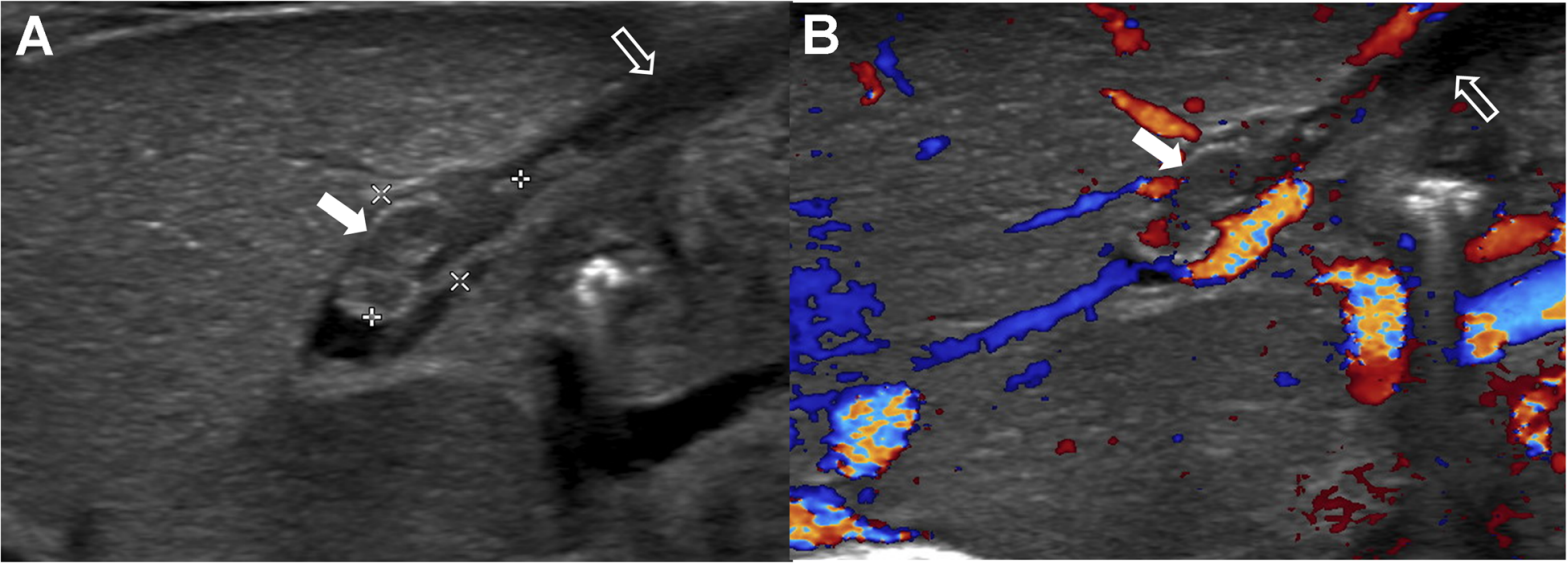
**a** Longitudinal gray scale sonogram of the left lobe of the liver on postnatal day 6 shows a thrombus (white arrow) within the umbilico-portal confluence of the left portal vein contiguous with the umbilical vein (open arrow) as a 1.10 × 0.60 cm-sized, hypoechoic tubular structure in two dimensions. **b** Corresponding color Doppler sonogram shows non-obstructive thrombosis (white arrow) with the presence of flow around the thrombus

**Fig. 2:**
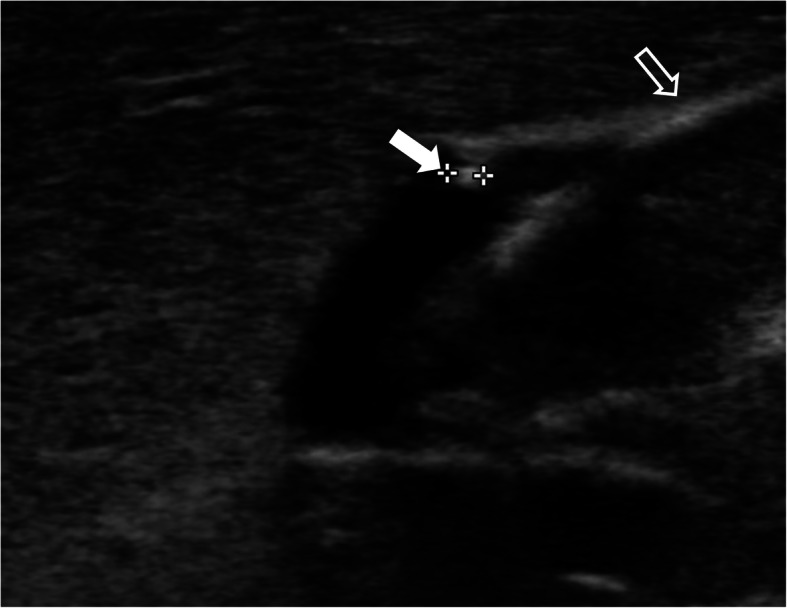
Longitudinal gray scale sonogram of the left lobe of the liver at the age of 6 months depicts partial resolution of the thrombus (white arrow) within the left portal vein, as the sonographic signal of the intraluminal thrombus decreased in size and became hyperechoic over time, with the adjacent ligamentum teres (open arrow) as a result of the involution of the umbilical vein via fibrotic transformation

A Korean male infant with a weight of 2980 g (91st percentile) was born to a 37-year-old woman (gravida 2 and para 2) at 34^+ 5^ weeks’ gestation via normal spontaneous vaginal delivery. Apgar scores were 8 and 9 at 1 and 5 min after birth, respectively. The mother and other family members had no known allergies and no other particular medical history including the maternal history of diabetes, hypertension, dyslipidemia, and neurologic disease, with the mother’s consumption of a small amount of cow’s milk only as food additives during pregnancy. The pregnancy was not complicated by any abnormalities encompassing maternal fever, gestational diabetes, preeclampsia, premature or prolonged rupture of membranes, and placental abruption. The placental examination was unremarkable. The infant’s general condition was good and oral intake commenced with a total of 60 ml of preterm formula milk. Four hours after initial feeding, three episodes of non-projectile, non-bilious emesis developed with approximately 5 ml milky vomitus on each occasion, followed by hematochezia 5 h later. Table 1 shows the flow of the infant’s laboratory and clinical data. Initial laboratory evaluation demonstrated a mild leukocytosis (white blood cells 11,400/μL) and eosinophilia (eosinophil fraction 14% and eosinophils 1530/μL), raised D-dimer 1718.3 vs. control < 500 ng/mL DDU, and mild conjugated hyperbilirubinemia (total bilirubin 1.5 mg/dL with the direct fraction 40%), with the other following data (hemoglobin 13.0 g/dL, hematocrit 37.7%, platelets 274,000/μL, C-reactive protein 0.04 mg/dL, prothrombin time 13.4 vs. 10.1–12.6 s with the international normalized ratio 1.19 vs. 0.93–1.13, partial thromboplastin time 42.8 vs. 23.6–31.1 s, antithrombin III 43%, and fibrinogen 256 vs. 180–350 mg/dL). Serum total immunoglobulin E (IgE) was 0.1 IU/ml (normal values < 5.0) and specific IgEs to cow’s milk proteins (CMPs) were negative. The Apt test for fresh blood in the stools did not change color and fecal occult blood was positive. A chest-abdomen plain radiograph showed a normal bowel gas pattern without other abnormalities. Oral feeds were discontinued from postnatal day 1. Total parenteral nutrition was instituted on day 2 and maintained until day 9. The stools mixed with macroscopic bleeding more than in the form of spots persisted during the first 3 days of life. From day 5, oral feedings were reintroduced with breast milk without maternal diet modification and/or casein hydrolyzed milk, which were tolerable without reactions including gastrointestinal symptoms and metabolic acidosis. On day 6, abdominal ultrasonography revealed a non-obstructive thrombus within the umbilico-portal confluence of the left portal vein as a hypoechoic tubular structure (Fig. 1a and b). On day 19, the peak eosinophil count of 3790/μL was observed with a low serum 25-hydroxyvitamin D 16.1 ng/mL (insufficiency < 30 and deficiency < 10). On day 75, the eosinophil count decreased to 590/μL and a supervised oral challenge to cow’s milk was performed. Feeding intolerance with vomiting and blood-tinged stools was noted and the feeding was changed back to the casein-hydrolyzed formula. At 6 months of age, oral re-challenge to regular cow’s milk feeding was attempted and no reaction was observed. The sonographic signal of the intraluminal thrombus gradually decreased in size and became heterogeneously hyperechoic over time (Fig. 2). At 1 year of age of the last follow-up ultrasonography, the thrombus completely resolved. The infant showed good weight gain with well-tolerated formula feeds.

**Table 1:** Laboratory and clinical data of the present case

Serum parameters	Day 1	Day 6	Day 13	Day 19	Day 33	Day 75	1 year
Hemoglobin (g/dL)	13.0	12.5	10.9	11.0	9.5	9.4	12.3
Hematocrit (%)	37.7	35.1	30.6	29.8	26.0	27.5	35.4
White cell count (/μL)	11,400	11,570	13,700	15,800	10,300	7800	9540
Eosinophil fraction (%)	14	16	11	24	15	7	4
Eosinophils (/μL)	1530	1800	1560	3790	1630	590	380
Platelets (/μL)	274,000	292,000	470,000	574,000	485,000	390,000	309,000
C-reactive protein (mg/dL)	0.04	0.4	0.17	–	–	–	–
Total bilirubin (mg/dL)	1.5	9.3	4.4	3	1.5	–	0.2
Direct bilirubin (mg/dL)	0.6	1.7	1.6	1.5	0.8	–	0.1
Direct fraction (%)	40	18	36	50	53	–	50
AST (22–71 IU/L)	18	19	18	22	22	–	40
ALT (10–40 IU/L)	4	7	10	10	10	–	20
25(OH) VitD (ng/mL)	–	–	–	16.1	–	20.9	26.4
Sonographic findings of the LPV thrombus	–	1.10 × 0.60 cm-sized, hypoechoic	0.80 × 0.30 cm-sized, hypoechoic	0.65 × 0.20 cm-sized, hyperechoic	–	0.36 × 0.13 cm-sized, hyperechoic	Completely resolved
Clinical notes	Emesis (on day 1 only) and hematochezia (for 3 days) after MF, thereafter, NPO until day 4	No GI Sx (from day 4) TPN (from day 2 to day 9) BF (from day 5)	No GI Sx BF + CHMF (from day 10)	No GI Sx BF + CHMF	No GI Sx BF + CHMF	Emesis and hematochezia after supervised OCMC, thereafter, recommencing CHMF	MF + CF since the successful OCMC at 6 months of age

*AST* Aspartate transaminase, *ALT* Alanine transaminase, *25(OH) VitD* 25-Hydroxyvitamin D, *LPV* Left portal vein, *MF* Cow’s milk-based formula feeding, *NPO* Nil per os, *TPN* Total parenteral nutrition, *GI Sx* Gastrointestinal symptoms, *BF* Breast feeding, *CHMF* Casein hydrolyzed milk feeding, *OCMC* Oral cow’s milk challenge, *CF* Complementary feeding

## Discussion and conclusions

Our premature infant presented with moderate-to-severe eosinophilia or hypereosinophilia (eosinophil counts > 1500/mm^3^) [6], which had been detected over at least 1 month after birth (Table 1). The hypereosinophilia was initially observed prior to the first formula feeding and may be associated with the two concurrent conditions in the newborn period: FPIAP as a CMA manifesting as bloody stools without typical allergic cutaneous and respiratory symptoms following a brief exposure to CMP and grade 1 PVT according to the proposed grading system by Morag et al. [17], appearing as non-occlusive portal venous thrombus with normal liver parenchyma at ultrasound.

FPIAP is a benign non-IgE-mediated food hypersensitivity, mostly as CMP intolerance, presenting in the first 6 months of life with blood-mixed stools in well-looking infants, generally with the remission by 1 year of age [18, 19]. In this case, the diagnosis of FPIAP was established based on the clinical history including CMP intolerance symptoms (emesis and hematochezia after the initial feeding), recovery from the symptoms after elimination diets, and the confirmatory challenge (recurrence of the symptoms following oral provocation with cow’s milk) despite lack of rectosigmoidoscopy with a biopsy to document the histopathology of eosinophilic proctocolitis. The other causes of rectal bleeding including necrotizing enterocolitis and food protein-induced enterocolitis syndrome were discriminated via the findings including mild symptoms, the infant’s otherwise healthy status, the C-reactive protein levels and non-specific radiographic results. The CMA of our infant resulted in being self-limited since the successful oral cow’s milk re-challenge at 6 months of age, with the normalized eosinophil count.

Neonatal PVT is a rare thrombotic event incidentally found during routine evaluation for other diseases in infancy [20]. A prospective study of 123 premature neonates born at < 33 weeks’ gestation or with < 1500 g birthweight revealed the high PVT incidence of 43% [21]. In the majority of cases, neonatal PVT affects the left portal vein encompassing umbilico-portal confluence, is clinically silent, is associated with UVC, particularly inadequate peripheral placement of catheter tips under the diaphragm, and shows favorable short-term outcomes with spontaneous resolution of the most thrombi without anticoagulant or fibrinolytic therapy [16, 20, 21]. However, serious complications including liver lobe atrophy, portal hypertension and gastrointestinal hemorrhage can develop later in childhood largely in persistent, extensive and occlusive PVT [20, 22]. A recent prospective case-control study on thrombosis in the UVC route with serial ultrasound screening did not demonstrate any thrombus forming process in 20 matched control neonates at 27–41 weeks’ gestation without UVC until median day 13 (1–17) after birth [16]. In our infant, PVT occurred with no history of central venous catheterization as a single lesion, non-occlusive and not propagating, within the left portal vein, which was fortuitously identified in the ultrasonography and completely resolved with expectant management. Accordingly, our case is in line with previous reports addressing that for the treatment of neonatal thrombosis, a ‘watch and wait’ approach may be applicable to most cases, especially cases with asymptomatic thrombi [16, 21].

Eosinophils have been believed to be possible direct target cells in cellular immune responses induced by CMP [23]. A recent study of 185 infants with FPIAP documented that eosinophil counts > 1000/mm^3^ were associated with severe rectal bleeding with mild-to-moderate anemia [18]. Eosinophils may be directly involved in thrombogenesis via several molecules in the intracytoplasmic specific granules with effects associated with thrombin generation and fibrin formation, including major basic protein (MBP), eosinophilic cationic protein (ECP), eosinophil peroxidase (EPO) and the coagulation initiator tissue factor [11, 12]. These molecules may lead to tissue and endothelial cell damage through cytotoxic effects, can inhibit the anticoagulant and fibrinolytic pathways, and can enhance activation of platelets and the tissue factor (extrinsic) coagulation pathway as shown in the various following mechanisms: 1) MBP and ECP neutralize negatively charged heparan-sulphate and exogenous heparin that bind to antithrombin by themselves or stimulating platelet factor 4 release, leading to failure to hinder factor X activation and thrombin generation [24, 25]; 2) MBP, ECP and EPO block anionic thrombomodulin that mediates thrombin dependent-activation of protein C with inhibitory effects on factor V and factor VIII [26, 27]; 3) MBP, ECP and EPO inhibit factor XII that can activate fibrinolysis [28]; 4) Eosinophils produce platelet-activating factor [29] and activate platelets along with MBP and EPO as strong platelet agonists [30]; 5) Eosinophils release tissue factor that activates factor X via factor VII activation and can stimulate tissue factor expression of endothelial cells via EPO [31]; 6) Eosinophils express CD40 ligand and contribute to initiation and progression of thrombosis [32]. In our infant, the development of PVT in the situation without UVC may be a result of the common intersection of multiple factors including hypereosinophilia, the immature hemostatic system of preterm newborn susceptible to thromboembolism [15], and hemodynamic changes by the adjacent ductus venosus involution after birth [16]. The initial eosinophilia before oral feeding may be from congenital conditions including immune responses to foreign antigens such as CMP, leading to sensitization in utero. The postnatal exposure to CMP may cause the reactive eosinophilia to be prolonged and more severe. During the period of hypereosinophilia, the FPIAP and PVT findings improved solely with CMP avoidance and conservative management (Table 1). This suggests that the eosinophilia in this case may have limited or complementary roles on the pathophysiologies of FPIAP and PVT, as part of the background environment related to symptom development of these disorders.

In numerous prior reports, venous and/or arterial thromboembolism is found associated with peripheral blood eosinophilia, mostly hypereosinophilia that shows abnormally high eosinophil counts and lasts for longer than 6 months, in a variety of disorders including idiopathic hypereosinophilia without organ damage, hypereosinophilic syndrome, Churg-Strauss syndrome and parasitic diseases [10–12]. Transient eosinophilia less than 6 months of duration has also been reported related to venous thromboembolism presenting with multivessel involvement including portal venous system in teen-aged and adult patients [13, 14, 33–35]. The hypereosinophilia of these patients was idiopathic (without a primary cause) or secondary to conditions leading to eosinophilia, ranged from 1600 to 9350 cells/mm^3^ of absolute eosinophil counts, and was largely associated with thrombocytopenia, remitting with resolved thrombi after multimodal treatments including anticoagulants, thrombolytics, and corticosteroids [13, 14, 33–35]. The current case is the first reported newborn case and precisely depicted alterations of the eosinophilia and the portal venous thrombus during the disease course (Table 1) representing a natural course of PVT and hypereosinophilia in a premature infant with conservative management. However, in our case, the lack of data on inherited or acquired thrombophilic risk factors including protein C and S deficiencies, factor V Leiden mutation, prothrombin G20210A mutation and fibrinogen receptor mutation (PLA2 polymorphism of the platelet membrane glycoprotein IIIa gene) should be considered in data interpretation. For the development of neonatal venous thromboembolism with multifactorial etiology, inherited thrombophilia is a serious risk factor, which, however, weighs less heavily on the prothrombotic scale than clinical factors [36]. Previous studies have shown extremely low thrombotic risk in otherwise healthy children with a single identified thrombophilic defect, no difference in the prevalence of inherited thrombophilia between neonates with venous thromboembolism and the healthy population [36], and the presence of two and greater genetic defects with protein S deficiency as a leading cause of recurrent venous thromboembolism [37].

Consequently this case suggests that in the setting of hypereosinophilia with a prolonged duration in premature neonates, FPIAP should be suspected in case of hematochezia in otherwise healthy infants, and considering the increased thrombotic risk by the prolonged hypereosinophilia and premature newborn status, evaluation for neonatal thrombosis may be needed, including PVT with the potential risk for the more serious, but uncommon, late complications encompassing portal hypertension.

## Data Availability

All data generated or analysed during this study are included in this published article.

## Abbreviations

CMA: Cow’s milk allergy
CMP: Cow’s milk protein
FPIAP: Food protein-induced allergic proctocolitis
IgE: Immunoglobulin E
PVT: Portal vein thrombosis
UVC: Umbilical venous catheterization
